# Comparative analysis of DNA collection techniques applied to disposable 3-layer earloop face masks

**DOI:** 10.1093/fsr/owaf041

**Published:** 2026-02-05

**Authors:** Niramon Masuntisuk, Tayawee Romgaew, Natcha Sanpang, Sunisa Aobaom

**Affiliations:** Biology and DNA Sub-Division, Central Police Forensic Science Division, Office of Police Forensic Science, Bangkok, Thailand; Biology and DNA Sub-Division, Central Police Forensic Science Division, Office of Police Forensic Science, Bangkok, Thailand; Biology and DNA Sub-Division, Central Police Forensic Science Division, Office of Police Forensic Science, Bangkok, Thailand; Division of Forensic Science, Department of Medical Technology, Faculty of Allied Health Sciences, Thammasat University Rangsit Campus, Pathum Thani, Thailand

**Keywords:** forensic sciences, face mask, DNA collection method, forensic DNA evidence, quantitation DNA

## Abstract

Since COVID-19 has emerged and become a global health issue, an awareness of wearing face masks has been attentive to prevent the spread of the disease. Face masks that have become a part of daily life, may be encountered at crime scenes and serve as a potential source of DNA for human identification. This study developed a rapid method for obtaining abundant DNA from 3-layer disposable face masks. Ten healthy volunteers were recruited to wear the masks for a period of 2 h. The optimal method for retrieving DNA was determined to be direct cutting of the middle section of the mask’s inner layer. The average DNA concentrations from males and females were 0.127 0 ± 0.233 7 ng/μL and 0.069 9 ± 0.107 4 ng/μL, respectively, with no significant difference observed between the sexes (*P* = 0.286). Furthermore, testing a smaller area (28 cm^2^) from the mouth-covering region still yielded sufficient DNA for STR genotyping (average 0.025 5 ± 0.021 8 ng/μL). Comparison of the inner and the filter layers revealed that the filter layer contained significantly less DNA (average 0.021 3 ± 0.020 3 ng/μL, *P* < 0.05). A mockup study was conducted on two female volunteers using six commercial brands of medical, carbon, and super 3D mask types. The results showed that the minimum DNA concentration (0.008 3 ± 0.003 1 ng/μL) extracted from a carbon type is also plenteous for short tandem repeats (STR) genotyping. Finally, mockup samples with low copy number were genotyped and they produced profiles with >19 autosomal STR loci. This suggests that the method was suitable in DNA analysis when a face mask is found as forensic DNA evidence.

## Introduction

Due to the rapid transmission of COVID-19, face masks are highly recommended to prevent its spread. Despite the fact that the World Health Organization (WHO) has declared an end to the COVID-19 public health emergency and the number of infected patients has steadily declined, communities in Thailand continue to wear face masks because of severe air pollution, specifically from PM2.5. An effect of wearing a face mask is friction-induced transmission of skin cells or saliva secretions sticking to it. When touching object, biological substances such as skin cells or body fluid (saliva, sweat, and sebum) may leave on the object. DNA obtained from such biological substances is called “touch DNA” [1]. Shedder status, which is one of the important factors affecting the amount of touch DNA, has a correlation with sebum secretion level, and the faces have a sebum-rich areas [1–4]. Thus, DNA retrieved from face masks collected as evidence can be categorized as touch DNA. As a result, it can serve as critical crime scene evidence for identifying an individual.

**Figure 1:**
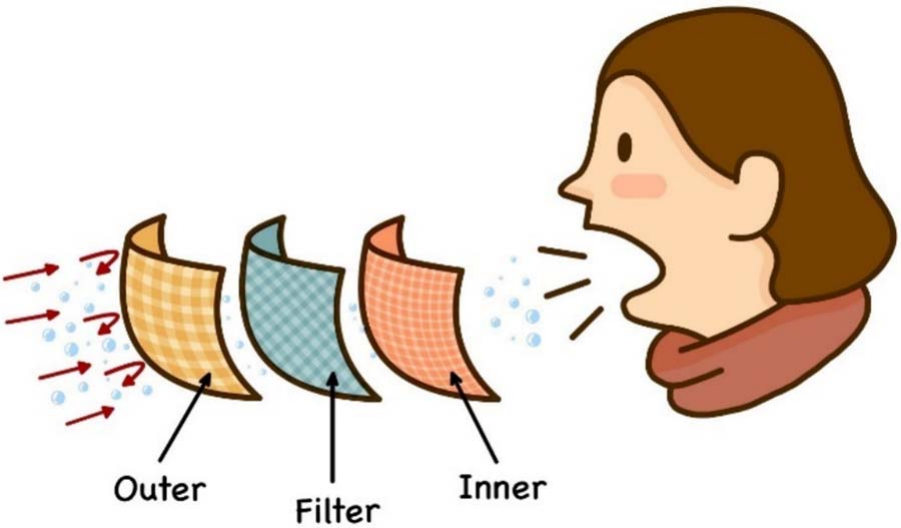
Layer composition of a three-layer face mask.

The most effective type of face mask in preventing infection and air pollution is the N-95, but because of its high cost and shortage during the pandemic, surgical/medical masks are more affordable and widely available, whilst still being highly efficient in both situations [5, 6]. Generally, a medical face mask consists of three layers: the outer layer, which acts as a shield against respiratory droplets and is water-repellent; the filter layer in the middle, which blocks particles and secretions; and the inner layer, which is the innermost sheet in direct contact with the wearer’s skin and serves as a key component in controlling the escape of the wearer’s droplets (Figure 1). The inner layer is characterized by its hydrophilic properties and filtration efficiencies derived from a tightly woven cotton fabric, facilitating the absorption of splashed secretions from the wearer. Conversely, the filter and outer layers are designated to be hydrophobic, featuring reduced pore dimensions. In this multilayer configuration, a spunbonded non-woven or loosely woven fabric serves as the outer layer, whilst a melt-blown non-woven layer is strategically positioned in the middle. This structural design not only minimizes the pressure drop across the mask but also enhances its water repellency, thereby contributing to protection against environmental secretions [7]. There are three types of surfaces in forensic science, including (1) porous surface (small pore surface which materials can penetrate), (2) non-porous surface (no pore consisted on surface), and (3) semi-porous (porous surface covered with another layer) [8]. As a result, a face mask may also be considered a semi-porous substrate that can act as a repellant and a collector of the wearer’s respiratory droplets.

A previous study [9] proved that wearing a face mask for at least 2 h can yield a full human DNA profile. Furthermore, Dong et al. [10] studied approaches to recover touch DNA from varied surfaces. Their results demonstrate that the direct cutting method is suitable for porous surfaces, whilst vacuum sweeper method is suitable for non-porous surfaces. Furthermore, the adhesive tape method can recovered touch DNA with a sufficient yield, comparable to the direct cutting method. Additionally, it can prevent the destruction of crucial evidence during processing [11].

Based on previous research that explored techniques for recovering touch DNA from various surfaces, this study focused on identifying an appropriate method for collecting touch DNA samples from three-layer disposable ear-loop masks. Four different methods—double swabbing, tape lifting, direct cutting of face mask layers, and cutting the ear-loops—were compared to determine the most suitable one for acquiring a sufficient amount of DNA for identification.

## Material and methods

### Mask samples preparation

The masks used in this study were three-layer disposable ear-loop types. Each mask was worn by a healthy volunteer for 2 h during routine daily activities. All volunteers provided informed written consent. After use, the masks were stored overnight in a light-free environment at room temperature. Study protocols were approved by the Human Research Ethics Committee of Thammasat University (Science), Thailand, in accordance with the compliance to the Declaration of Helsinki, the Belmont report, CIOMS guidelines and the international practice (ICH-GCP) (COA No. 162/2565).

### DNA collection from mask samples

**Figure 2:**
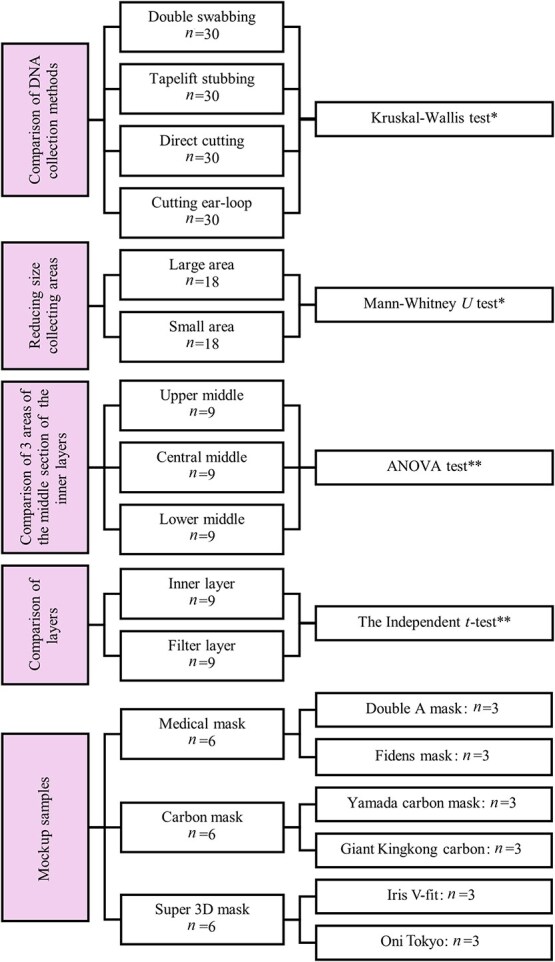
Sample size allocation and statistical approach as determined by data suitability. ^*^Statistical analysis for non-parametric data. ^**^Statistical analysis for parametric data.

This study included multiple replicates for each processing, as illustrated in Figure 2. The statistical methods were selected based on the nature and distribution of the data. A detailed explanation of each step is provided below.

#### Comparison of DNA concentrations obtained using four methods

The inner layer of a face mask, with a total area of 84 cm^2^ (6 cm × 14 cm), was vertically divided into three sections: left, middle, and right. Samples were collected from the middle section using one of three methods: direct cutting, which involved excising the middle section of the inner layer with clean scissors; double swabbing, which used a wet swab (Thai Gauze, Bangkok, Thailand) followed by a dry swab (Thai Gauze) on the same area; and tape lifting, which employed adhesive tape (3M™, Bangkok, Thailand) to stub the defined area. Moreover, a fourth method, cutting the ear-loop, involved cutting the entire ear-loop pieces with clean scissors. Each sample was collected in a 2 μL tube for the lysis and incubation steps.

This study involved 10 healthy volunteers (five males and five females), aged 18 years or above, without oral complications or ongoing dental procedures. From each volunteer, three masks were allocated to each of the four DNA collection methods. This design yielded a total of 120 mask samples yielded a total of 120 mask samples (three masks per volunteer, 10 volunteers for each method, four methods). DNA was extracted and quantified from all samples to compare concentrations between males and females and to identify the most effective collection method for face mask evidence.

#### Comparison of DNA concentrations obtained using the best DNA collection method on reduced areas

**Figure 3:**
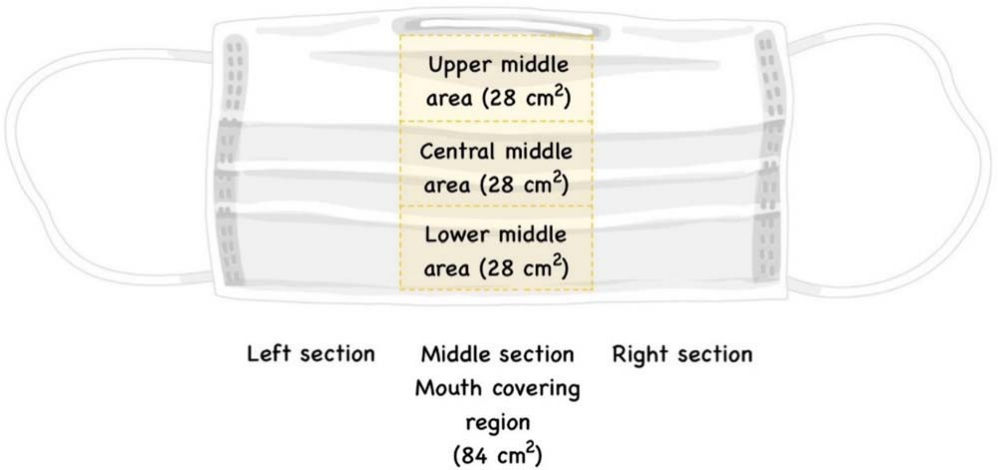
Layer of three-layer disposable ear-loop mask. Highlighted area is the middle section of the inner layer which was divided horizontally into three small areas studied in Section *Comparison of DNA concentrations obtained using the best DNA collection method and reduced areas on different layers*.

In this study, DNA concentrations between large-area and reduced areas were compared using a sample set comprising three face masks from each of two volunteers. The collection method determined to be most effective in a prior experiment (detailed in Section *Comparison of DNA concentrations obtained using four methods*) was employed to collect DNA from the middle section of the inner layer (measuring 84 cm^2^). This section was subdivided horizontally into three distinct areas of 28 cm^2^: the upper middle region (designated as the nasal area for touch DNA), the central middle area (pertaining to salivary secretions), and the lower middle region (identified as the chin area for touch DNA) (Figure 3). Each sample was collected in a 2 μL tube for lysis and incubation.

#### Comparison of DNA concentrations obtained using the best DNA collection method and reduced areas on different layers

To analyze DNA concentrations derived from various layers of face masks, additional DNA samples totaling nine were collected from the filter layer worn by a single volunteer as investigated in Section *Comparison of DNA concentrations obtained using the best DNA collection method on reduced areas.*

#### DNA collection from mockup mask samples

This study included commonly used face masks, namely medical masks, carbon masks, and 3D masks, as samples to assess the performance of previously studied methods in obtaining sufficient DNA for human identification. Three mask samples of each type were examined. The optimal DNA collection method, as determined in Section *Comparison of DNA concentrations obtained using four methods* was employed, along with the specific location and face mask layer investigated in Sections *Comparison of DNA concentrations obtained using the best DNA collection method on reduced areas* and *Comparison of DNA concentrations obtained using the best DNA collection method and reduced areas on different layers*.

### DNA extraction

All DNA samples were extracted by QIAamp DNA Mini Kit protocol (Qiagen, Sunnyvale, CA, USA). Before starting, the samples were added 400 μL phosphate-buffered saline (PBS), 200 μL Buffer AL and 20 μL Proteinase K and equilibrated to 56°C for 1 h. The samples were eluted with volumes of 30 μL elution buffer [12].

### Quantification of DNA

All DNA extracts were quantified by half-reaction Investigator Quantiplex Kit (Qiagen) on Quanstudio 5 (Thermo Fisher Scientific, Waltham, MA, USA). Collected DNA concentrations were analyzed by Design & Analysis 2.6.0 Real-Time PCR system (Thermo Fisher Scientific).

### Direct STR profiling

DNA extracts from mockup face mask samples exceeding 0.01 ng/μL in initial concentration were amplified *via* a half-reaction Globalfiler™ IQC PCR Amplification Kit on 3500xL Genetic analyzer (Applied Biosystem, Foster City, CA, USA). Subsequently, short tandem repeat typing was analyzed by GeneMapper® IDX v.1.4 (Applied Biosystem).

### Statistical analysis

All experimental data were subjected to statistical analysis using SPSS version 29 (IBM, Armonk, NY, USA) and were classified as either parametric or non-parametric. The specific statistic methods applied to each data type are summarized as below (Figure 2).

Parametric data: Independent *t*-test (for two groups); ANOVA (for more than two groups). Non-parametric data: Kruskal-Wallis test (for more than two groups; applied when data from any group were non-parametric). In all tests, a *P-*value of ˂0.05 was considered statistically significant, denoting a difference at the 95% confidence interval (CI).

## Results

### Methods for DNA collection on three-layer disposable ear-loop face mask

**Figure 4:**
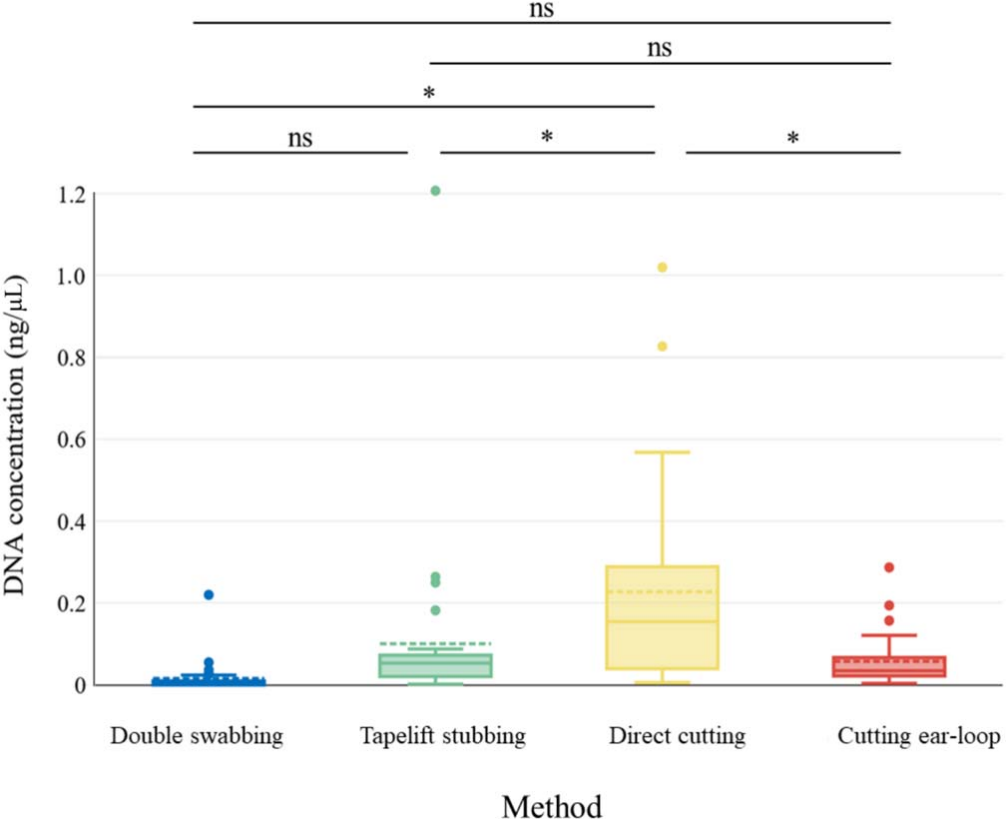
DNA concentration of four collection methods: double swabbing, tape lifting, direct cutting on the middle section of the inner layer and direct cutting on the ear-loop fabric. Mean, median, standard deviation, and the highest/lowest concentrations are included.

Direct cutting of the inner layer’s middle section yielded the highest average DNA concentration (0.227 3 ± 0.242 9 ng/μL), whereas swabbing method showed the lowest (0.008 2 ± 0.013 4 ng/μL) (Figure 4, Table 1). The DNA concentrations from four collection methods were compared by one-way ANOVA (with Tukey’s HSD post-hoc test) and the Kruskal-Wallis test. The results demonstrated that cutting method applied to the mouth-covering region produced a significantly higher DNA yield than the swabbing, tape lifting, and ear-loop cutting methods.

**Table 1:** Descriptive statistic results and DNA concentration (ng/μL) mean ranking from four DNA collection methods and Kruskal-Wallis testing results between the four methods.

DNA collection methods	Mean ± SD	Median (range)	Mean rank	Ranking	*P-*value
Double swabbing inner layer (84 cm^2^; *n* = 30)	0.008 2 ± 0.013 4	0.002 4 (0.000 0–0.054 8)	22.47	4	0.000^a^
Tape-lifting inner layer (84 cm^2^; *n* = 30)	0.100 7 ± 0.218 3	0.053 1 (0.001 9–1.206 8)	67.93	2	0.000^a^
Cutting inner layer (84 cm^2^; *n* = 30)	0.227 3 ± 0.242 9	0.154 5 (0.005 1–1.019 6)	87.20	1	0.000^a^
Cutting ear-loop (whole pieces; *n* = 30)	0.057 6 ± 0.061 2	0.034 2 (0.004 3–0.287 0)	64.40	3	0.000^a^

SD: standard deviation.

^a^ *P* < 0.05 is considered statistically significant.

In this study, significant Kruskal-Wallis test results identified the direct cutting method from the middle section of the inner layer—a porous substrate—as the best method for collecting DNA from face masks. This method achieved the highest mean rank in DNA concentration (87.20) (Table 1). Therefore, it was selected as the standard DNA collection method for all subsequent investigations in this research.

### Reduced areas and layer composition of DNA collection

#### Reducing size collecting areas on face masks

DNA was quantified from 24 large-area samples (84 cm^2^ of the middle section of the inner layer) and 18 small-area samples (28 cm^2^ of three areas on the middle section of the inner layer). A Mann–Whitney *U* test revealed a statistically significant difference in DNA concentration between large- and small-area samples from both male and female volunteers (*P* < 0.05, 95%CI). Though the average DNA concentration of large-area samples was higher, the yield from small-area samples (0.025 5 ng/μL) was sufficient to serve as a template for human DNA identification (0.008 ng/μL) [13, 14]. Therefore, an area of 28 cm^2^ of the middle section of the inner layer could be the minimum area for subsequent studies of our research.

#### DNA concentration from three areas of the middle section of the inner layer

DNA extracted from three areas (28 cm^2^) of mouth-covering region of the inner layer were quantified. The average DNA concentrations of upper middle, central middle, and lower middle areas were 0.034 0 ± 0.017 1, 0.029 0 ± 0.022 0, and 0.014 0 ± 0.009 6, respectively (Table 2). The maximum DNA concentrations of upper middle, central middle, and lower middle areas were 0.066 0 ng/μL, 0.070 0 ng/μL, and 0.032 0 ng/μL, respectively, whereas the minimum concentrations were 0.0140 ng/μL, 0.002 0 ng/μL, and 0.003 0 ng/μL, respectively. The ANOVA test revealed that DNA concentrations from these small areas were not significantly different (*P* > 0.05, 95%CI). The middle section of the inner layer is mouth-covering region. It was shown that there was no difference between collected DNA concentrations of the reduced areas of this region. Therefore, reduced areas of at least 28 cm^2^ total area may be required.

#### DNA concentration from inner layer and filter layer of the middle section of three-layer disposable ear-loop face mask

A total of 18 samples (each 28 cm^2^) were collected from the upper, central, and lower areas of the middle section of a face mask from one female individual, comprising nine samples from the inner layer and nine from the filter layer. DNA was extracted from all samples and quantified. The average DNA concentrations for the inner and filter layers were 0.021 3 ± 0.020 3 ng/μL, and 0.002 1 ± 0.002 5 ng/μL, respectively, with the maximum DNA concentrations of 0.061 7 ng/μL and 0.006 9 ng/μL, respectively, and the minimum DNA concentrations of 0.001 9 ng/μL and 0.000 0 ng/μL, respectively (Table 3). The Independent *t*-test revealed a significant difference in DNA concentration between the two layers. Although the porous inner layer allows for droplet penetration, it also constitutes a direct contact surface with the face [13], which explains its significantly higher DNA compared to the filter layer. This finding suggests that the filter layer may not serve as an adequate substitute for the inner layer when seeking to avoid highly contaminated surfaces. Another area of the face mask is therefore recommended, i.e., ear-loop fabric.

### DNA concentration and genotyping on mockup mask sample

**Figure 5:**
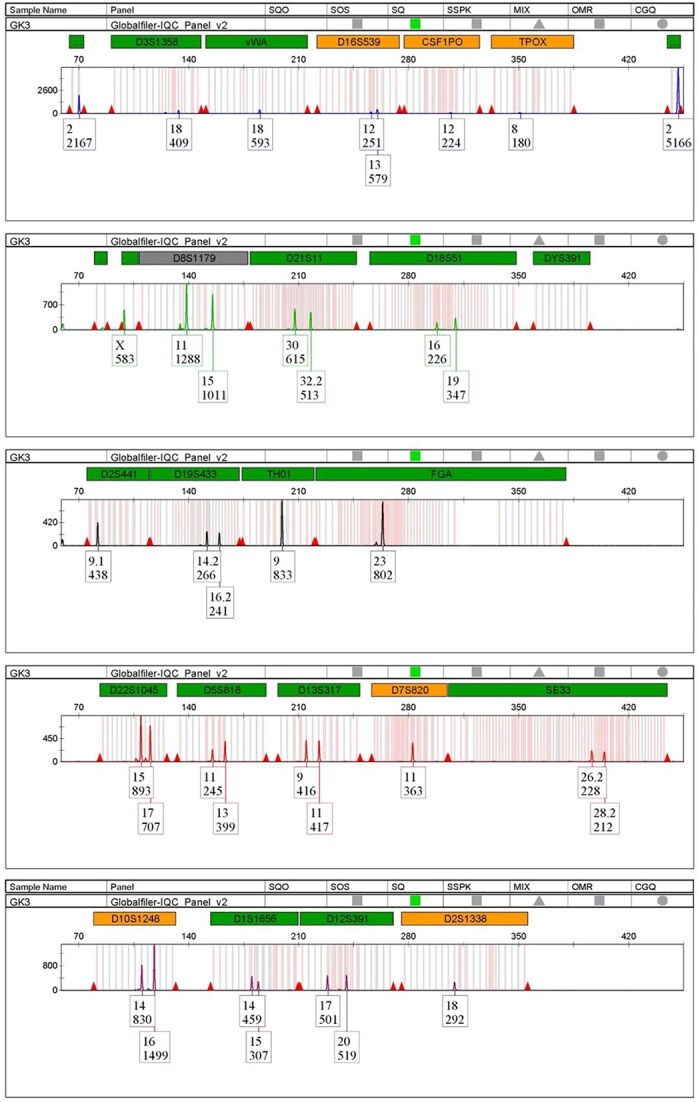
STR profile analysis from the lowest DNA concentration (0.004 0 ng/μL) extracted from Giant Kangkong carbon mask.

Based on results above, the central area of the middle section of the inner layer was selected for DNA collecting using the direct cutting method from mockup mask samples. Two commercial brands from each of the following types were tested: medical masks (Double A, Thailand; Fidens, Thailand), carbon masks (Yamada, Thailand; Giant Kingkong, Thailand), and Super 3D masks (Iris V-fit, Japan; Oni Tokyo mask, Japan). Three samples were collected from each brand. DNA extracted from the resulting 18 samples were quantified (Table 4). The maximum DNA concentration was obtained from the Oni Tokyo mask samples (0.416 3 ng/μL), whilst the minimun was from the Giant Kingkong carbon mask (0.008 3 ng/μL). Despite being the lowest concentration, 0.008 3 ng/μL of DNA was sufficient to generate a full STR profile using the Globalfiler™ IQC PCR Amplification Kit and the Powerplex® Fusion 6C System [11]. DNA Samples with concentrations below 0.01 ng/μL were subjected to autosomal STR genotyping. The results showed that these samples can produce profiles with over 19 autosomal STR loci (Table 5, Figure 5).

**Table 4:** Average DNA concentration of mockup mask samples.

Mask type	Brand	Average DNA concentration ^a^(ng/μL)
Medical mask	Double A mask	0.073 7 ± 0.051 6
	Fidens mask	0.026 0 ± 0.011 8
Carbon mask	Yamada carbon mask	0.334 7 ± 0.344 8
	Giant Kingkong carbon mask	0.008 3 ± 0.003 1
Super 3D mask	Iris V-fit mask	0.023 0 ± 0.011 3
	Oni Tokyo mask	0.416 3 ± 0.192 7

^a^DNA concentrations <0.01 ng/μL is considered statistically significant.

**Table 5:** Number of autosomal STR loci of <0.01 ng/μL DNA from mockup mask samples.

Mask type	Brand	DNA concentration (ng/μL)	Number of autosomal loci
Medical mask	Double A mask	0.008 0	19
Carbon mask	Yamada carbon mask	0.006 0	19
	Giant Kingkong carbon mask	0.004 0	21

These results show that the DNA collection method selected in this study is an effective technique for processing face mask evidence.

## Discussion

The objective of the study was to determine the optimal method for collecting DNA from face masks. Four techniques were evaluated: double swabbing, tape lifting, direct cutting of the mask, and cutting the ear loop. Our findings indicate that the direct cutting method yielded the highest DNA quantity. This result is consistent with prior studies which have also demonstrated the superior yield of the cutting technique [11, 15]. Moreover, the direct cutting method has been established as the most effective approach for collecting DNA from various porous materials such as cloth, gloves, and thin ropes [10]. In contrast, the double swabbing and tape lifting methods yielded significantly lower DNA concentrations. This aligns with previous research on brick surfaces, where swabbing was most successful and tape lifting was least effective [15].

This research also compared DNA yields from areas directly contacting facial skin and mouth to those from the ear loops. The results confirmed that the former provided significantly higher DNA quantities than the ear loop areas. This observation aligns with previous research comparing the mouth-covered area to ear loops, which also reported higher DNA concentrations from the mouth area [7]. Additionally, previous studies have noted DNA concentration variations between these areas without a significant impact on obtaining a usable DNA profile [16].

When studying the factors in DNA transfer, it was determined that DNA sampling is influenced by numerous factors [17]. Firstly, Jansson et al. [1] demonstrated a strong association between an individual’s shedder status and the level of DNA accumulation on the facial skin, hence the individual’s secretion level is a factor in DNA transfer.

Secondly, the duration of mask wear significantly impacts DNA yield. In this study, a 2 h wearing period was sufficient to recover quantifiable DNA. This aligns with previous research in which DNA was detectable after just 2 h of wearing, with the amount increasing over time and the initial 2 h period being adequate for DNA profiling [9]. In addition, Dash et al. [18] detected DNA concentration on cloth masks, N-95 mask type and surgical disposable masks with 4 cm^2^ area with direct cutting method. Although their DNA concentration results were higher, wearing time of mask was longer at least ~12 times (1 day). Their results showed that DNA yield depended on wearing time.

Thirdly, the specific sampling location on the mask is a key determinant. For practicality, we reduced the sampling area to one-third of its original size. It was revealed that the middle section, covering the nose, mouth, and chin, yielded the highest DNA concentration. A more detailed analysis of this section showed that the area corresponding to the mouth (the saliva secreting region) produced the most DNA. Therefore, the precise location on the mask is one determinant that could impact the quantity of DNA obtained. Furthermore, factors influencing DNA deposition, such as activity, surface type, friction, and moisture influence the deposition, persistence, and transfer of DNA, contributing to the heterogeneity in DNA recovery [3, 4, 17].

**Table 2:** Comparison of DNA concentrations collected from three small areas of the middle section of the inner layer by ANOVA testing.

Location	DNA concentration (ng/μL)		
	Maximum	Minimum	Mean ± SD
Upper middle	0.066 0	0.014 0	0.034 0 ± 0.017 1
Central middle	0.070 0	0.002 0	0.029 0 ± 0.022 0
Lower middle	0.032 0	0.003 0	0.014 0 ± 0.009 6
*P-*value^a^	0.267		

SD: standard deviation.

^a^ *P* < 0.05 is considered statistically significant.

**Table 3:** Comparison of DNA concentrations collected from the inner layer and the filter layer by the Independent *t*-test.

Location	DNA concentration (ng/μL)		
	Maximum	Minimum	Mean ± SD
Inner layer	0.061 7	0.001 9	0.021 3 ± 0.020 3
Filter layer	0.006 9	0.000 0	0.002 1 ± 0.002 5
*P-*value^a^	0.001 0		

SD: standard deviation.

^a^ *P* < 0.05 is considered statistically significant.

Furthermore, our analysis of different mask layers confirmed that the inner layer, designed to absorb secretions [19], was the most effective for DNA collection.

Upon analyzing the experimental results to assess a mockup surgical mask, a minimum of eight loci of autosomal STR are required for human identification [20]. It was observed that the samples could generate profiles with at least 19 autosomal STR loci. Similar to other studies, the DNA concentration obtained from the filter mask component was low, as compared with other sources such as toothbrushes and razors. Nonetheless, it was still sufficient to obtain comprehensive DNA profiles [16].

## Conclusion

The results showed that the best method for collecting DNA from a three-layer disposable ear-loop face mask was the direct cutting of the middle section of the inner layer from a reduced DNA collecting area. The inner layer accumulated more DNA than the filter layer, and gender showed no effect on DNA concentration from masks. Moreover, a requisite amount of DNA was successfully detected from mockup samples of face mask brands commonly found in the market in Thailand using the selected methods and area.

These results indicated that direct cutting of the central middle area (28 cm^2^) of the inner layer could be a rapid and suggested technique for DNA collection from three-layer disposable ear-loop face mask evidence in forensic DNA investigation.

The research highlights an intriguing aspect for further study: reducing the size of the mask sample and accounting for intrinsic elements such as dirt or moisture, which can interfere DNA analysis. These factors are commonly encountered in everyday life, and further research is warranted to refine DNA extraction techniques under these conditions.
